# Effect of Bone Mineral Density on Rotator Cuff Tear: An Osteoporotic Rabbit Model

**DOI:** 10.1371/journal.pone.0139384

**Published:** 2015-10-14

**Authors:** Xiaobin Chen, Hugo Giambini, Ephraim Ben-Abraham, Kai-Nan An, Ahmad Nassr, Chunfeng Zhao

**Affiliations:** 1 Biomechanics Laboratory, Division of Orthopedic Research, Mayo Clinic, Rochester, Minnesota, United States of America; 2 Institute of Orthopaedics, Chinese PLA, Beijing Army General Hospital, Beijing, China; 3 Department of Orthopedic Surgery, Mayo Clinic, Rochester, Minnesota, United States of America; 4 Department of Radiology, Mayo Clinic, Rochester, Minnesota, United States of America; Queen Mary University of London, UNITED KINGDOM

## Abstract

**Introduction:**

An increased bone mineral density (BMD) in the proximity to tendon insertion can improve rotator cuff repair and healing. However, how a decrease of BMD in the humeral head affects the biomechanical properties of the rotator cuff tendon is still unclear. Previous studies have demonstrated ovariectomy in animals to lead to osteoporosis and decreased BMD, and Teriparatide (PTH) administration to improve BMD and strength of bone. This study aimed to explore the correlation between humeral head BMD and infraspinatus (ISP) tendon insertion strength, and if an increase in bone quantity of the humeral head can improve the strength of the rotator cuff.

**Materials and Methods:**

Eighteen New England white rabbits were divided into the 3 groups: Control, Ovariectomy-Saline (OVX-Saline), and Ovariectomy-PTH (OVX-PTH). The OVX-Saline group and the OVX-PTH were administered daily saline and Teriparatide injections for 8 weeks starting at 17 weeks of OVX. BMD of the humeral head was measured, the ISP tendon failure load was tested and the failure stress was calculated. One specimen from each group was used for histological analysis. Linear regression analysis was used to derive equations for the BMD and failure stress.

**Results:**

Significant differences were observed in the measured humeral head BMD of the Control and OVX-PTH groups compared to the OVX-Saline group (P = 0.0004 and P = 0.0024, respectively). No significant difference was found in failure stress among the three groups, but an expected trend with the control group and OVX-PTH group presenting higher failure strength compared to the OVX-Saline group. BMD at the humeral head showed a positive linear correlation with stress (r^2^ = 0.54). Histology results showed the superiority in OVX-PTH group ISP enthesis compared to the OVX-Saline group.

**Conclusion:**

Bone loss of the humeral head leads to decreased tendon/bone insertion strength of the infraspinatus tendon enthesis. Teriparatide administration can increase bone density of the humeral head and may improve the mechanical properties of the infraspinatus tendon enthesis.

## Introduction

Tears of the rotator cuff tendon, which are common in patients over 60 years old, often cause shoulder pain and limited shoulder function [1]. Neer et al. [2] described that a tear of the rotator cuff is followed by atrophy of the rotator cuff muscles and weakness of external rotation and abduction, leading to inactivity and a decreased range of active motion, leaking of the synovial fluid, and instability of the humeral head. This in turn results in both nutritional and mechanical factors that cause disuse osteoporosis of the humeral head. Some clinical studies have also shown osteopenic changes in the greater tuberosity in patients with chronic rotator cuff tears, and this finding was attributed to the loss of physical stimuli at the tendon insertion point, in accordance with Wolff’s law [3, 4].

For patients with rotator cuff tear, the significance of the bony status at the tendon insertion and in the greater tuberosity has been well recognized [2, 5]. An increased bone mineral density and trabecular structure in the greater tuberosity and in the proximity to the insertion site can improve rotator cuff repair and healing. Chung et al. [6] found bone mineral density to be one of the independent factors predicting rotator cuff healing. Similarly, Charousset et al. [7] found healing to occur more likely in patients with good bone quality compared with those with poor quality. Does a decrease in bone density of the humeral head make it more likely for a rotator cuff to tear? A study by Jiang et al. using human cadaveric shoulders showed rotator cuff tears to be associated with loss of trabecular bone volume and trabecular connectivity in the greater tuberosity [8]. However, it is still unclear how degenerative changes on bone of the humeral head affect the biomechanical properties of the rotator cuff tendon and a possible occurrence of tear. Therefore, the objective of this study was to explore if increasing the bone quantity of the humeral head can improve the strength of the rotator cuff insertion and decrease the likelihood of a rotator cuff tear in patients with osteoporosis. For this purpose, we used an osteoporotic rabbit model and evaluated the effect of teriparatide (Parathyroid Hormone) as a bone anabolic agent on the infraspinatus (ISP) insertion failure strength. We hypothesized that teriparatide, by stimulating bone formation [9], would enhance the strength of the rotator cuff insertion and potentially decrease the likelihood of a rotator cuff tear in those with osteoporosis.

## Materials and Methods

### Animal Groups

Animal care and experimental procedures were conducted with the approval of the Institutional Animal Care and Use Committee of Mayo Clinic. Eighteen 10-month-old female New England white rabbits were used in the study and divided into the following groups: *Control* (n = 6, normal rabbits), *Ovariectomy-Saline* (*OVX-Saline*, OVX and daily saline injections) (n = 5, one rabbit died during surgery), and *Ovariectomy-PTH* (*OVX-PTH*) (n = 6, OVX and daily Teriparatide injections). A postmenopausal osteoporotic model induced by OVX was performed as previously described and raised for 17 weeks [10]. The surgical rabbits received routine pain control for 2 days postoperatively and were raised in the Mayo Clinic animal laboratory. Before the OVX procedure and 17 weeks after, bone mineral density (BMD) was measured using dual energy x-ray absorptiometry (DEXA) (Lunar iDXATM, GE Healthcare, USA) to confirm bone density reduction and the development of osteoporosis. Post 17 weeks, the OVX-Saline group and the OVX-PTH were administered daily saline and 10μg/kg/day Teriparatide injections for 8 weeks.

The rabbits in each group were euthanized under general anesthesia using 3cc of Pentobarbital IV (FatalPlus) and bilateral shoulders were dissected and stored at -20°C. In preparation for testing, the shoulders were thawed at room temperature and dissected free from all other muscular, capsular, and ligamentous constraints, leaving the ISP tendon-bone complex. Specimens were kept moist with normal saline solution during all phases of dissection, preparation, and testing.

### Bone Mineral Density Measurements

Bone mineral density (BMD) was measured using dual energy x-ray absorptiometry (DEXA) analysis (Lunar iDXATM, GE Healthcare, USA). All humeri were placed in the same position with the condyle in contact with the equipment screen to ensure similar orientation and placement of the specimens. A uniform region of interest (ROI) of 68 x 83 pixels was set in each specimen. The ROI selection was based on previous observations of the size of the humeral head and chosen to include most of the humeral head trabecular region.

### Anesthesia Protocol

Bone density measurements were carried out under short general anesthesia consisting on intramuscular injections of Ketamine (35mg/kg), Xylazine (5mg/kg). During the surgical OVX procedure, the anesthesia was maintained using Isoflurane 1.5–3% with oxygen.

### Biomechanics test

The humerus was transected 1cm distal from the inferior border of the infraspinatus (ISP). The ISP was then cut 3 cm proximal from the inferior border of the insertion site. In an effort to minimize soft tissue slippage during mechanical testing, a pony tail suture (Ethicon Inc., Cincinnati, OH) was placed in the ISP tendon, about 0.8 mm away from the insertion site, carefully preserving the tendon-bone interface. Width and thickness were measured at the insertion site using a precision caliper with 0.1 mm accuracy. Cross-sectional area was then computed by assuming an oval cross section.

**Fig 1:**
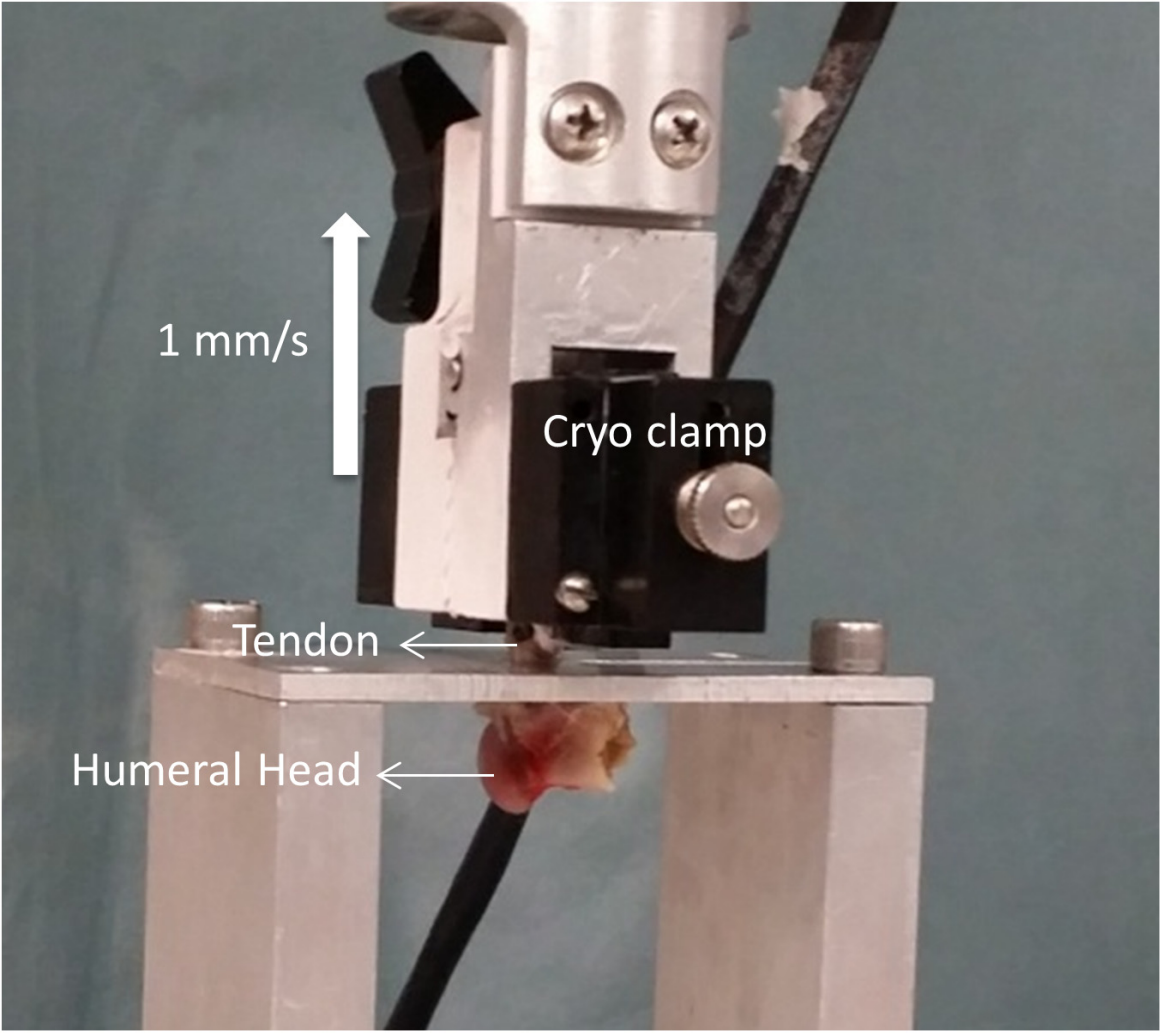
The humerus was placed in a custom made fixture and the tendon attached to a cryo-clamp for testing. Failure was tested at 1 mm/s and the direction of pull of the tendon was directly superior and along the axes of the ISP tendon fibers.

A servo-hydraulic testing machine (MTS Systems Corporation, Minneapolis, MN) was used to test the tendon insertion strength. The humerus was placed in a custom made fixture that allowed the bony part to freely move while the ISP tendon was pulled superiorly along the axes of the tendon fibers (Fig 1). The tendon with the pony tail suture was rigidly fixed in a cryogenic clamp about 0.5–1 cm away from the insertion. The tendons were pulled at a rate of 1 mm/s, and the load at failure was recorded for each tendon. Mode of failure was recorded by a single observer by direct observation of the specimen during testing. Failure stress was then calculated by normalizing the measured failure loads to the computed cross-sectional area.

### Histological examination

One specimen from each group was left intact for histological analysis. Specimens were fixed with 4% paraformaldehyde overnight, decalcified with 15% EDTA, and embedded in optical cutting temperature compound (Tissue-Tek, Sakura Finetek). Coronal sections were cut at 10-μm thickness using a cryostat (Leica CM 1850, Wetzlar, Germany) and stained with hematoxylin and eosin (H&E). Insertion site morphology was qualitatively observed under light microscopy. Sections were analyzed using an optical microscope to assess the overall histologic structure and microstructure of the ISP enthesis, such as the 4-layered structure, including the tendon, the non-mineralized and mineralized fibrocartilage and bone, and the tidemark, which forms a boundary between two fibrocartilages, as previously reported [11].

### Data Analysis

All data are expressed as means ± SD (standard deviation). One-way analysis of variance (ANOVA) and Student-Newman-Keuls (SNK) test were performed to compare differences between groups. Linear regression analysis was used to derive equations for the BMD and failure stress using SPSS 12.0 (SPSS, Chicago, IL, USA). Differences were considered to be significant when *P* < 0.05.

## Results

After sparing one specimen (shoulder) in each group for histology, there were a total of eleven specimens in the control group, nine specimens in the *OVX-Saline* group, and eleven specimens in the *OVX-PTH* group for the biomechanical testing. Table 1 summarizes all the obtained data. No significant difference was found in the cross-sectional width, thickness and area between the three groups.

**Table 1:** Summary of morphological data and experimental outcomes.

	*Control*	*OVX-Saline*	*OVX-PTH*
Cross-section width (mm)	3.55±0.10	3.57±0.05	3.57±0.06
Cross-section thickness (mm)	1.57±0.08	1.56±0.07	1.55±0.08
Cross-section area (mm^2^)	4.38±0.28	4.36±0.22	4.33±0.25
Humeral Head BMD (g/cm^2^)	0.394±0.027*	0.338±0.027	0.386±0.031**
Ultimate Stress (MPa)	32.34±5.99	25.27±8.50	32.37±8.92

Different from OVX-Saline:

* *P = 0*.*0004*

** *P = 0*.*0024*

### BMD and Failure Load Analysis

**Fig 2:**
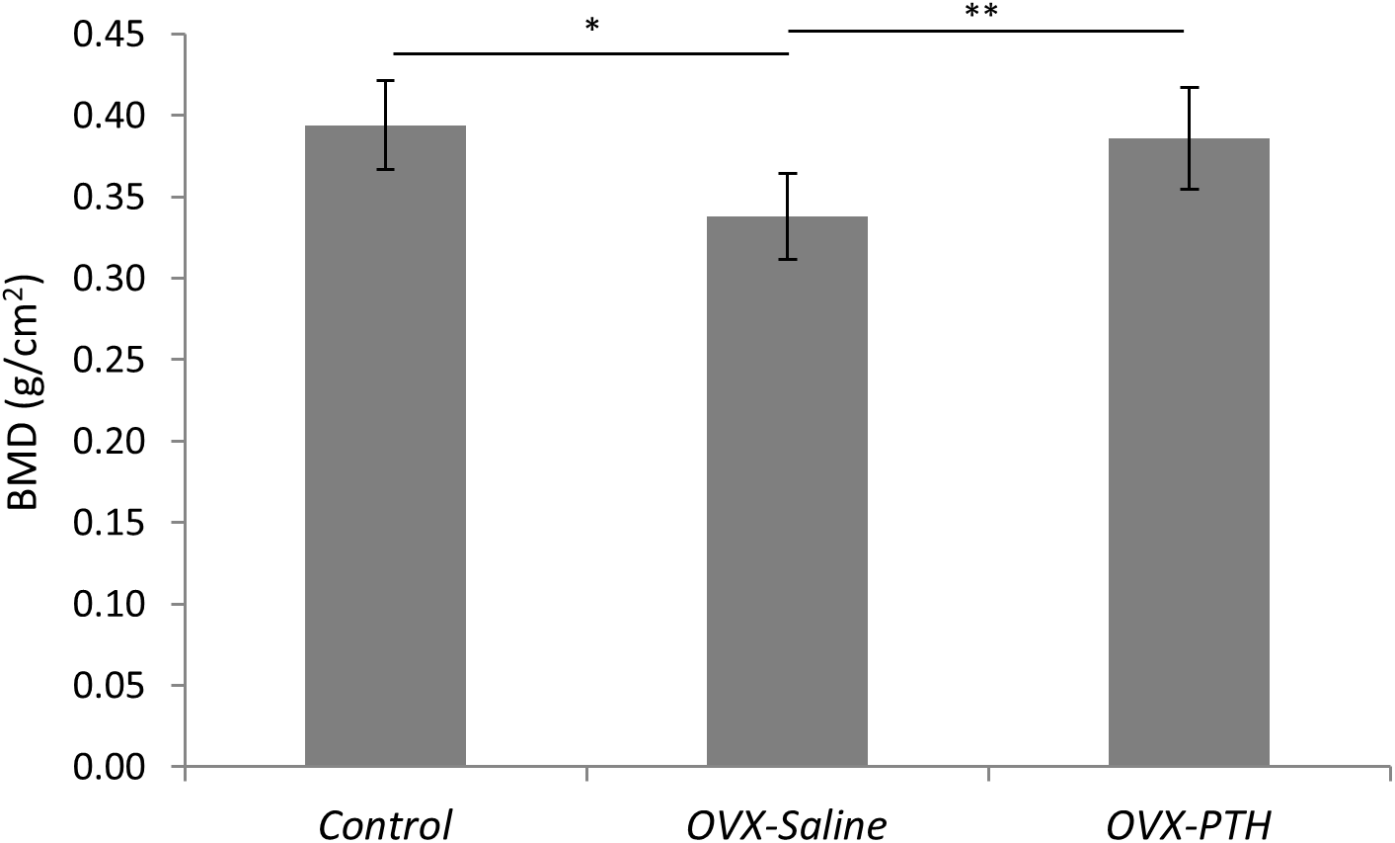
Significant differences were observed in bone mineral density of the humeral head between the *Control*, *OVX-Saline* and *OVX-PTH* groups. Bone mineral density in the *OVX-PTH* group was significantly higher than in the *OVX-Saline* group but similar to that of the *Control* group.

**Fig 3:**
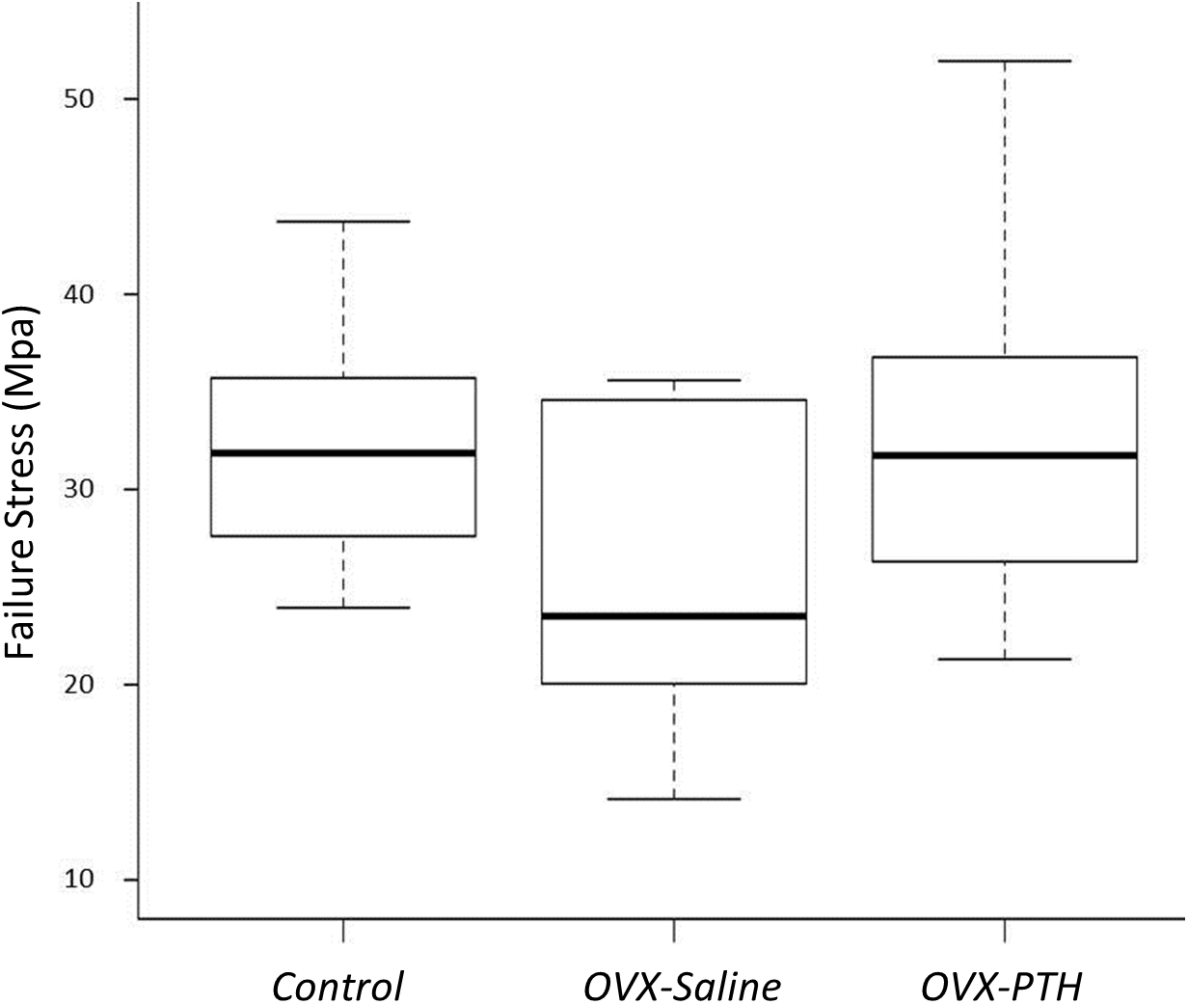
Failure stress for all groups. Although not significant, a visual trend can be observed where the *OVX-Saline* group presents smaller failure loads when compared to the *Control* and *OVX-PTH* specimens. This shows the effect of PTH administration in increasing failure strength and potentially preventing rotator cuff tears.

**Fig 4:**
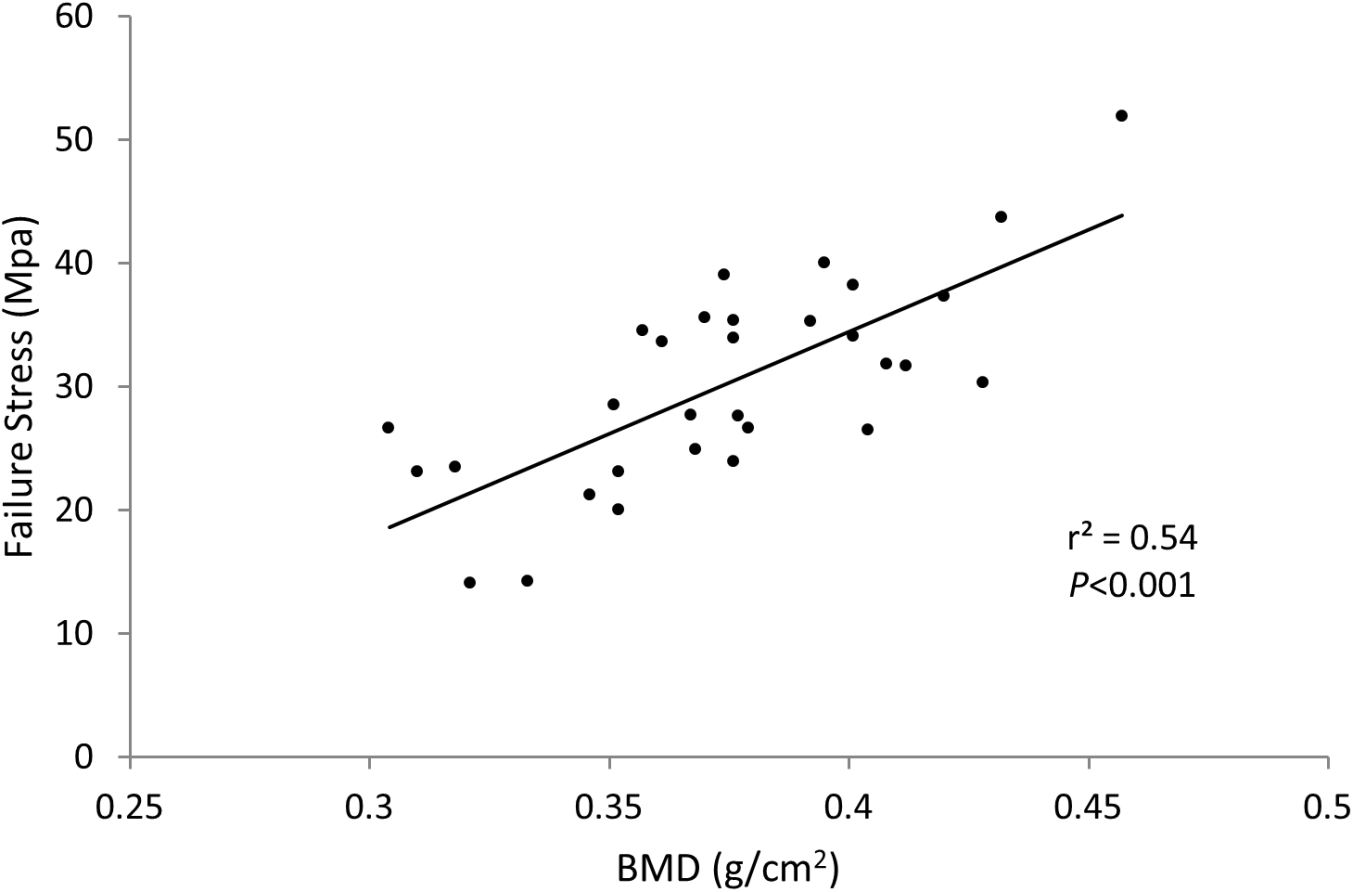
Failure Stress vs. BMD. A positive linear correlation was observed between failure stress and bone mineral density measured at the humeral head.

BMD of the *Control* and *OVX-PTH* groups was significantly higher than that of the *OVX-Saline* group (*P* = 0.0004 and *P* = 0.0024, respectively) (Table 1 and Fig 2). There was no significant difference in BMD between *Control* and *OVX-PTH* groups. Although no significant difference was found in the ultimate stress between the three groups, there is an expected trend in failure load and stress with the control group exhibiting higher failure strength than the *OVX-Saline* group, and the *OVX-PTH* group presenting similar strength values as controls and higher failure strength than the *OVX-Saline* rabbits (Table 1) (Fig 3). Bone mineral density at the humeral head showed a positive linear correlation with stress (r^2^ = 0.54, *P*<0.001) (Fig 4). During mechanical testing, all infraspinatus tendons failed at bone/tendon insertion site.

### Histology

**Fig 5:**
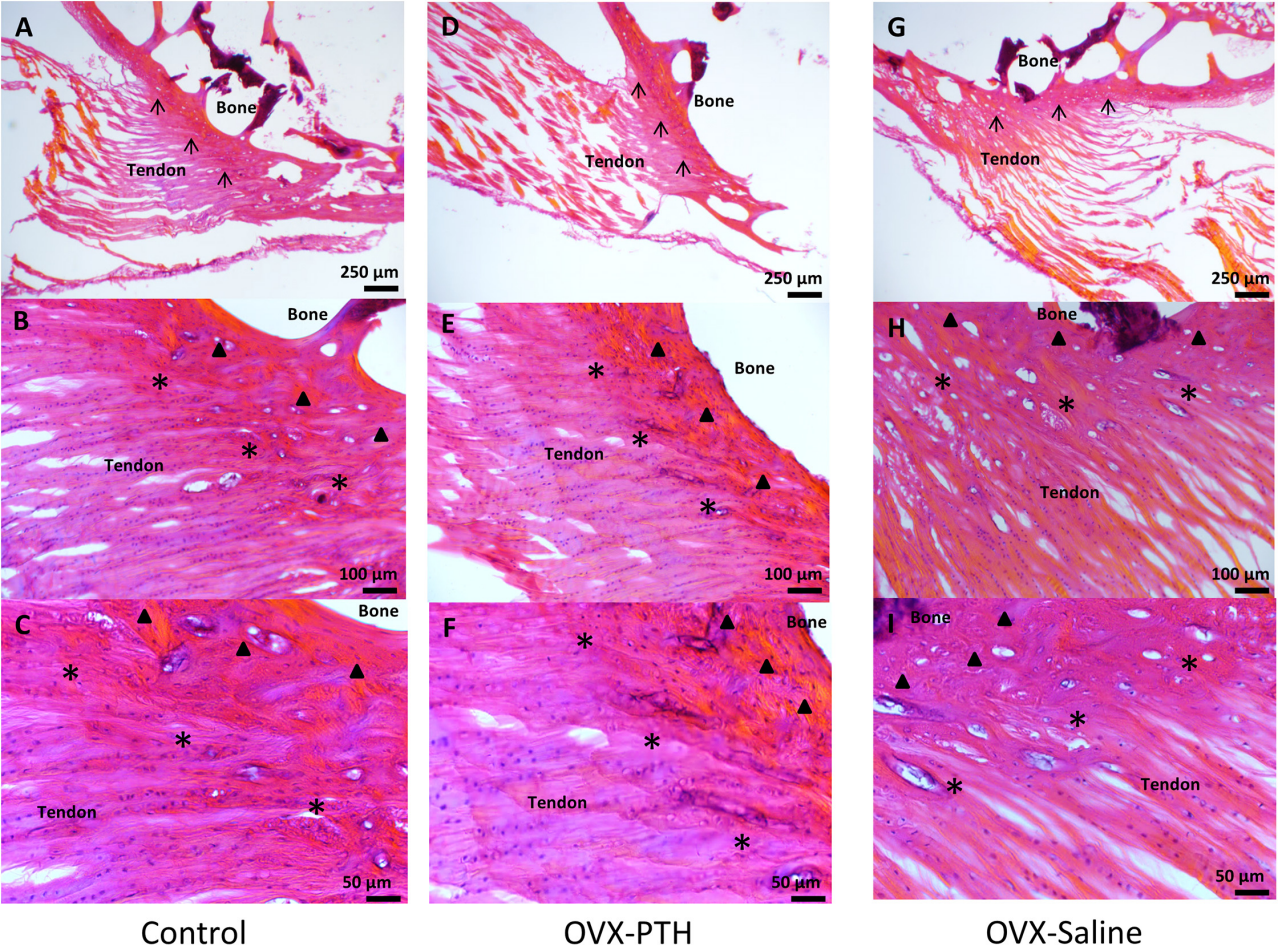
4-layered tendon-bone interface of ISP enthesis: tendon, nonmineralized fibrocartilage (*), mineralized fibrocartilage (▲) (▲), and bone. (↑) indicates the tidemark. In the *Control* and *OVX-PTH* groups, well-organized ISP enthesis could be observed with a clear tidemark (A, D). Arrangement of nonmineralized fibrocartilage aligned well, nonmineralized and mineralized fibrocartilage were stained deeply and with abundant cells (B, E). In the *OVX-PTH* group, the nonmineralized and mineralized fibrocartilage were thicker with a more compact structure (E, F). In *OVX-Saline* group, a relative thinner tendon-bone interface was observed, as well as a less clear tidemark in the enthesis (G). The cell deposition in the area was less compared to the other groups, and osteoclast number and bone lacuna in the mineralized fibrocartilage seemed to have increased compared to both *Control* and *OVX-PTH* groups (H, I).

The *Control* and *OVX-PTH* groups had a well-organized 4-layered tendon-bone interface of ISP enthesis (tendon, nonmineralized fibrocartilage, mineralized fibrocartilage, and bone). Their tidemarks were clear and there was a concise arrangement of nonmineralized fibrocartilage, as well as a deep staining with abundant cells of nonmineralized and mineralized fibrocartilage. More importantly, the *OVX-PTH* group showed nonmineralized and mineralized fibrocartilage to be thicker with a more compact structure. In contrast, the *OVX-Saline* group showed a relative thinner tendon-bone interface, the tidemark in the enthesis was not clearly observed, and the staining was not as strong when compared to the same area in the other groups. Although the arrangement of nonmineralized fibrocartilage still seemed to be aligned, the tissue seemed less compact and the amount of cell deposition in that area was smaller than in the other two groups. Furthermore, the mineralized fibrocartilage area of the *OVX-Saline* group showed an increased number of bone lacunae which can relate to an increased osteoclast activity and number in this group (Fig 5).

## Discussion

Rotator cuff tear is an aging-related degeneration leading to shoulder pain and dysfunction [12]. Although overuse injury has been considered as the major causation, the true etiology of the rotator cuff is not fully understood [13]. It has been generally recognized that BMD of the humeral head affect rotator cuff repair healing; the higher the BMD, the better the healing [6, 7]. A cadaveric study revealed that incidence of rotator cuff tear directly correlated to the humeral head BMD [8]. However, this correlation in clinical scenario has not been established. In this study, we measured the ISP tendon tensile strength at the insertion site using an osteoporotic rabbit model (OVX model), and further studied if teriparatide administration could improve bone mineral density of the humeral head in OVX rabbits undergoing bone loss, leading to an increased ISP tensile strength. Although osteoporotic rabbit models have been previously described, the resulting bone loss in the rabbit humeral head has not been clearly defined. In our study, compared to control rabbits, the OVX procedure caused a reduction in the humeral head BMD where the rotator cuff inserts. This suggest the current rabbit model of osteoporosis as a reliable model for ovarian deprivation osteoporosis, with the rabbit shoulder serving to investigate human rotator cuff and bone loss progression.

Teriparatide [human parathyroid hormone (PTH)(1–34)] is a new therapeutic option for osteoporosis that has been shown to induce new bone formation onto trabecular and cortical surfaces in monkeys, women, and men [14, 15]. Previous studies showed that PTH strengthens vertebra in monkeys and humans, resulting in reduced rates of vertebral fractures in postmenopausal women with osteoporosis [16, 17]. In addition, teriparatide reduced the risk of nonvertebral fragility fractures by 53–54% in a large, double-blind, placebo-controlled clinical trial [18]. Teriparatide had beneficial effects on the skeletal mass, structural architecture, and biomechanical integrity of the hip from OVX monkeys, despite increasing cortical porosity. However, the efficacy of teriparatide in the humeral head has not been explicitly demonstrated in the literature. The results of this study suggest that teriparatide administration, in a rabbit OVX model, can increase bone density in the humeral head where the rotator cuff inserts. Our current study showed that, with teriparatide treatment for 8 weeks with a daily subcutaneous dose of 10 μg/kg, BMD of the humeral head in the *OVX-PTH* group was significantly increased compared to the *OVX-Saline* group and nearly restored to the level of the control group. These results suggest that PTH administration may be beneficial for reducing the risk of rotator cuff tears or reducing the rate of the repair re-tears, which is a common complication following rotator cuff repair, especially for the osteoporotic patient population [19, 20].

The importance of the bony status at the tendon insertion and in the greater tuberosity has been well recognized in the management of patients with rotator cuff tear. Some clinical and basic science studies, using a rotator cuff injury and repair model, demonstrated that bisphosphonate treatment can improve bone mineral density at the rotator cuff footprint and enhance the biomechanical properties of rotator cuff repairs [2, 21, 22]. Most studies ascribe the osseous changes in the greater tuberosity observed following rotator cuff tears to both nutritional and disuse osteoporosis. However, it is possible that these changes might precede the tear itself, and the poor bone condition may be an important factor in causing rotator cuff tears. Wren et al. examined the influence of the bone mineral density in the mode by which human Achilles tendon fail [23]. Interestingly, they found that the older individuals, with lower BMD, were likely to suffer avulsions ruptures compared to younger individuals, with higher BMD, who were more likely to experience tendon ruptures. These results showed BMD as a key component in the mode of failure of the tendon-bone interface. Therefore, it was necessary to complement previous research findings by supporting the hypothesis that treatment to increase BMD in the humeral head may improve the biomechanical characteristics of the rotator cuff and help prevent a tear, such as in those with osteoporosis. We combined all three groups in this study to assess the linear correlation between BMD and stress, following anatomical measurements. The failure stress of the infraspinatus tendon increased with increasing BMD of the humeral head. Although not reaching a statistical significant result, the control and *OVX-PTH* groups showed higher mean failure stress values compared to those of the *OVX-Saline* group. This trend might become significant statistically if the sample size increased. Numerous studies have shown that the physical loading environment influences the formation of bone architecture and bone density [24–26]. Accordingly, a reduction of tendon-bone load can lead to severe functional defects in bone, tendon, and cartilage. On the other hand, Schwartz et. al [27] also demonstrated that a loss of bone and fibrocartilage would have a detrimental effect on the ability of the tendon enthesis to transfer forces from tendon to bone. This loss of mechanical function is due to disruption of collagen fiber structural organization and alterations in the composition of the mineralized interface. Our study showed consistent results between failure stress and histology outcomes. The *OVX-PTH* group had a well-organized tendon-bone interface of the ISP, and the structure of the non-mineralized and mineralized fibrocartilage was thicker and more compact when compared to the *OVX-Saline* group. This is consistent with the higher biomechanical properties of the tendon insertion.

In addition to changes in bone quality, hormone changes, such as estrogen deficiency via the ovariectomy procedure, or teripartide administration, might have an effect on tendon properties. It has been previously demonstrated that estrogen deficiency negatively affects tenocyte biosynthesis and leads to an increase in cell apoptosis, affecting the biomechanical properties of the tendon structure [28, 29]. Similarly, estrogen deficiency has been shown to negatively affect tendon metabolism and healing [30]. However, the effect of the absence of estrogen on the tendon enthesis still remains unknown. Both, tendon and bone properties, should synergistically affect tendon insertion strength. It is known that PTH enhances fracture healing, however, little is known about its effect in tendon. A previous study on tendon repair by Lee et al. demonstrated that PTH could increase the deposition of fibrous tissue which correlated to an increase in the expression of collagens and fibronectin [31].

There were several limitations in this study. First, although a visible trend is visible relating differences in failure stress between the groups, a small sample size was used which may have prevented us from reaching a statistically significant difference between them. Meanwhile, due to a lack of published data relating tensile failure strength at the tendon insertion in rabbits, a power analysis could not be performed to justify our sample numbers. However, we believe this pilot study can further our understanding relating the effect of bone mineral density on rotator cuff insertion strength, which has not been previously reported. Based on this encouraging preliminary data future studies should include a larger sample population with comparative interventions. Second, we used dual-energy x-ray absorptiometry (DXA) to measure bone mineral density (BMD) of the humeral head. Although this method has been previously shown to be a reliable technique in research and in clinical practice to measure BMD [32, 33], measurements of the greater tuberosity may have been masked and affected by the lesser tuberosity or the other structures of the proximal humerus that overlay the greater tuberosity. However, as we used a uniform standard to process DEXA and quantify the BMD, the results should be reliable. Third, tendon properties and changes due to the OVX procedure and treatment with PTH were not evaluated. Failure strength of the tendon insertion depends on the soft tissue properties as well as the bony insertion site. Future studies should also focus on analyzing quantitative molecular and tissue structural changes on the tendon and insertion site, associated with the absence of estrogen and the adminsitration of teriparatide.

In conclusion, estrogen deficiency induces bone loss of the humeral head and changes in the architecture of the infraspinatus tendon enthesis. Teriparatide administration can increase bone density of the humeral head and may improve the composition of the mineralized interface of infraspinatus tendon enthesis. These changes result in improved mechanical properties of the infraspinatus tendon enthesis. These results imply bone loss progression may be a risk factor for rotator cuff tears, and improving humeral bone density in patients with osteoporosis or osteopenia may help to enhance the rotator cuff strength and reduce the re-tear rate following rotator cuff repairs.

## Supporting Information

S1 File Data. (XLSX)
